# How water intermittency and water perceptions influence household coping strategies in northwestern Ecuador

**DOI:** 10.1371/journal.pwat.0000439

**Published:** 2025-09-22

**Authors:** Andrea Sosa-Moreno, Gwenyth O. Lee, James A. Trostle, Karen Levy, Josefina Coloma, Joseph N.S. Eisenberg

**Affiliations:** 1 Department of Epidemiology, University of Michigan, Ann Arbor, Michigan, United States of America,; 2 Rutgers Global Health Institute, Rutgers University, New Brunswick, New Jersey, United States of America,; 3 Department of Anthropology, Trinity College, Hartford, Connecticut, United States of America,; 4 Department of Environmental & Occupational Health Sciences, University of Washington, Seattle, Washington, United States of America,; 5 School of Public Health, University of California Berkeley, Berkeley, California, United States of America

## Abstract

Intermittent water supply (IWS) systems are characterized by water services that are unavailable for hours or days at a time, resulting in water insecurity even among households with piped water connections. While coping strategies for IWS are well-documented, their specific associations with measures of intermittency, such as weekly frequency, daily duration, and predictability of intermittent periods, are not well understood. Most existing research relies on descriptive comparisons across sites, with limited use of multivariable models to quantify these associations. To address these gaps, we used an explanatory sequential mixed-methods design to characterize household piped water intermittency and their association with coping strategies across three communities in northwestern Ecuador. In the quantitative phase, household surveys captured data on water supply and coping strategies for IWS. In the qualitative phase, semi-structured in-depth interviews explored resident’s perceptions of water access and quality. All three study communities experienced IWS, but the weekly frequency and daily duration of intermittent periods varied by location. Within Borbón, the largest town, neighborhood-level differences in IWS patterns were observed. After adjusting for household sociodemographic factors, each additional day per week without water supply was associated with a 30% higher odds of households treating their domestic water (1.30 [1.18–1.41]), and an 18% lower odds of relying on multiple drinking water sources (OR: 0.82 [0.72–0.92]). Each additional 3-hours per day without water supply was associated with a 12% higher odds of treating domestic water (OR: 1.12 [1.04 – 1.22]). Qualitative findings further suggested that household coping strategies were influenced by perceptions of water safety and quality, convenience, cost, and predictability. Unreliable water systems may erode trust in piped water, prompting households to adopt coping strategies that have their own risks and costs. Interventions that promote continuous water services and improved water management are essential to reduce health risks.

## Background

Water vulnerability remains a concern for many people worldwide [1–3]. UNICEF characterizes water vulnerability as a combination of water scarcity (limited presence of water) alongside a lack of access to drinking water services. Nearly half a billion children live in areas classified as having high or extremely high water vulnerability [3]. While global access to improved water systems has increased, from 3.5 billion people with access to piped water in 2000 to 5.3 billion in 2022, this access does not necessarily guarantee reliable and continuous water supply [4–6]. Over one billion people have access to a piped water system but live under conditions of intermittent water supply (IWS), characterized by water services that are unavailable for hours or days at a time [7]. The number of people dealing with IWS will likely increase in coming years due to water scarcity, intensified by climate change, infrastructure decay, and unplanned expansion of distribution networks driven by rapid urbanization [8–11]. Although water scarcity is a growing concern globally, water demand is expected to increase more rapidly in Sub-Saharan Africa and Latin America, highlighting the importance of understanding water-related challenges in these regions [3].

Lack of reliable access to safe and sufficient water is associated with adverse effects on health [12–14]. For example, individuals living in communities with IWS are at higher risk of diarrheal illness [7,8,15,16] or infection with specific enteric pathogens [17]. The risk of diarrheal disease can increase due to water contamination inside the piped water network [8,18]. It can also result from household-level contamination when families rely on coping strategies such as storing drinking water, which may be prone to recontamination [19–21]. Adverse health effects of inadequate access to water, sanitation, and hygiene (WASH) likely extend beyond just gastrointestinal illnesses. Households storing water may also be at higher risk of arboviral infection, since water containers can become breeding grounds for mosquitoes [22,23]. A recent meta-analysis observed an association between water insecurity and symptoms of common mental disorders, including depression and anxiety [13]. Moreover, the economic costs associated with poor water quality and limited water quantity can indirectly impact nutritional health outcomes [24]. For instance, households may spend a significant portion of their total income on purchasing bottled water or storage containers [25–27]. These increased water-related expenses have been associated with greater food insecurity [24,28], as they reduce the amount of money available for food purchases [29,30].

IWS usually occurs when water systems do not sufficiently meet user demand, or as a result of technical deficiencies, degradation over time, or unplanned expansions to the system [11]. When the water demand exceeds supply, pressure fluctuates, resulting in unsupplied areas, stagnation zones, and unreliable water supply. Inequities in water quality and water access [31,32] can be exacerbated as unreliable water tends to vary based on geographical location. Households located near treatment plants or primary pipelines typically experience shorter periods of IWS compared to those at the end of the distribution network due to increased demand, decreased pressure, and leakages [33–36]. These spatial disparities suggest that community-level assessments of intermittency may mask important within-community variation, highlighting the importance of capturing intermittency at the household-level. However, few studies to date have relied on household-level data to characterize IWS [37–40].

When a continuous 24-hour water supply is not feasible, guidelines for operating IWS systems recommend configurations in the water distribution based on community-driven service levels [36,41]. As a result, piped water intermittency can manifest differently depending on the weekly frequency (days/week without water supply) and daily duration (hours/day without water supply) of intermittent periods. For example, piped water may be available for long periods over a few days each week or shorter periods daily. Water supply may also be predictable or unpredictable [42], impacting the extent to which households can plan for, and respond to, water outages. Community-managed water systems, often proposed as alternatives to traditional government-management systems [43], have to make decisions about these trade-offs. Current research in WASH has focused on a binary definition of intermittency (i.e., whether a system is intermittent or not) [37]. For example, Trudeau, et al. used a binary indicator to assess water service interruptions, categorizing it as ‘reliable’ if the household experienced no interruptions, and ‘unreliable’ if there was at least one interruption during the month [44]. We were interested in addressing this gap by characterizing water intermittency using more nuanced measures of intermittency at the household-level.

Households utilize a variety of strategies to cope with water intermittency [45,46]. Some coping strategies include diversifying water sources, for example, purchasing water, borrowing from neighbors or increasing water storage. Others involve treating water to address perceived or actual contamination related to intermittency; or decreasing water usage through changes in household routines, hygiene, and diets [47–54]. These coping strategies lead to several health outcomes including malnutrition, gastrointestinal illnesses, and musculoskeletal injuries, particularly in women and children [55–58], as well as mosquito-borne diseases [14,46,59]. For example, storing water for long periods of time leads to microbial recontamination and subsequent gastrointestinal disease [19,20,60,61]. These coping strategies and other water-related behaviors can vary across settings depending on environmental, cultural and socioeconomic factors [46]. For example, studies in Guadalajara, Mexico and in our study site (Borbón, Ecuador), found increased prevalence of purchasing bottled water in response to IWS [47,62]. In contrast, households in Nyanza, Kenya did not purchase water, except in situations where women were unable to collect water themselves, such as during advanced pregnancy, early postpartum or periods of illness and fatigue [29].

Previous studies have compared coping strategies among settings with different IWS patterns. However, there has been little attention within the literature on the association between specific measures of intermittency (e.g., weekly frequency, daily duration, and predictability of intermittent periods) and coping strategies, particularly in small community-managed water systems. For example, Guragai, et al. found that communities with less than 4 hours/week of water supply were more likely to purchase tank-delivered water but less likely to treat piped water compared with communities with more than 8 hours/week of water supply [37]. Additionally, few studies to date have used regression models to quantify the association between household-level measures of piped water intermittency and coping strategies. DuChanois, et al., examined the use of multiple water sources and found it was associated with lower continuity of water services; however, their analysis was not limited to piped water sources [40].

In this study, we focused on understanding how household piped water intermittency impacts coping strategies. To achieve this, we conducted an explanatory sequential mixed-method design [63] to integrate results from quantitative and qualitative data in three communities in northwestern Ecuador that has long struggled with piped water access due to failed infrastructure projects and underinvestment. Our specific aims were to: 1) Quantify water intermittency across communities, focusing on weekly frequency, daily duration and predictability of intermittent periods; 2) Examine the association between water intermittency and coping strategies; and 3) Contextualize and interpret results from survey data through in-depth qualitative interviews with residents.

## Materials and methods

### Ethics statement

Participants provided their written informed consent. The protocol for the EcoDess project was reviewed and approved by ethical review committees at Universidad San Francisco de Quito (2017–159M) and the University of Michigan (HUM00140967). The analysis protocol for this study was reviewed by ethical review committees at Universidad San Francisco de Quito (2022–036M) and the University of Michigan (HUM00223929).

### Study site

The northwestern province of Esmeraldas in Ecuador has faced persistent issues with access to piped water, mainly due to decades of failed water infrastructure projects, lack of investment in public works, and corruption. Borbón (population ~7000), the region’s main commercial center, is connected by secondary roads with Maldonado (pop. ~ 2000) and Timbiré (pop. ~ 1000), located 20 and 40 minutes away, respectively (S1 Fig). Each community operates its own water plant through a community-managed committee responsible for water treatment and distribution. The water plant in Borbón was last renovated in 2006, followed by Maldonado in 2018 and Timbiré in 2020. Operators are residents of the community and are paid through the collection of household water fees. In Borbón and Timbiré, most households are equipped with water meters that determine usage-based charges. In contrast, Maldonado charges a flat monthly fee regardless of consumption. Periodic community meetings are held to discuss plant maintenance and issues related to water access. Treatment includes applying aluminum sulfate as a flocculant, and chlorine as a disinfectant and is intended to be applied daily prior to distribution, though actual availability may vary [47]. Chlorine levels are monitored occasionally in Borbón, every two days in Timbiré, and not at all in Maldonado. Plant maintenance routines also differ: sweeping and scrubbing of plant facilities occur every 8 days in Borbón, but only every two months in Maldonado and Timbiré. Storage tanks are cleaned monthly in Borbón and Timbiré, and every three months in Maldonado. Previous studies have suggested that variations in neighborhood elevation may influence water supply at our study site [64].

### Study design

**Fig 1.**
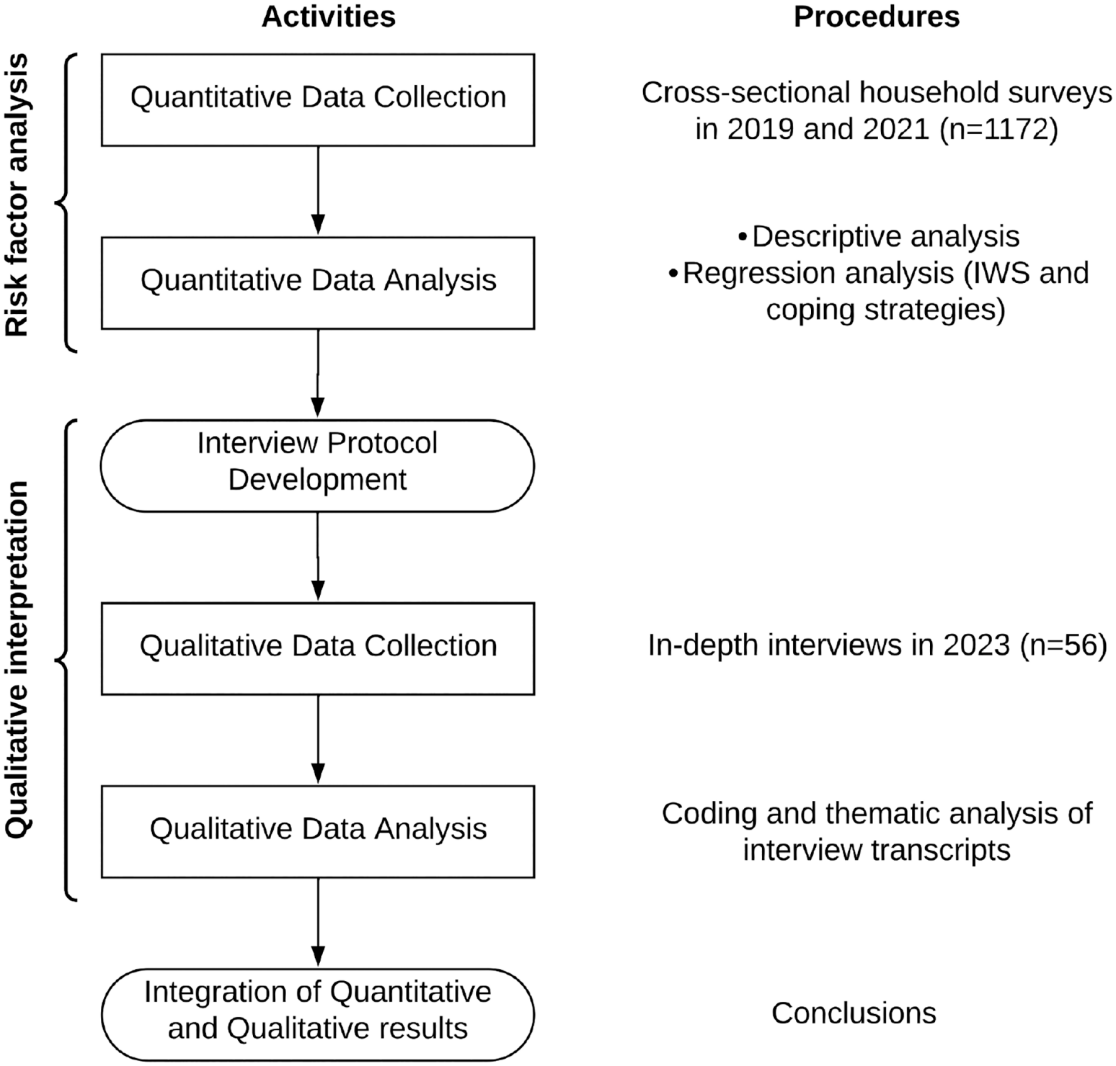
Visual diagram for our explanatory sequential mixed method design. Explanatory sequential mixed method design adapted from Ivankova and Stick (2007) [63]. IWS = Intermittent Water Supply. https://doi.org/10.1371/journal.pwat.0000439.g001

We used an explanatory sequential mixed-method design, characterized by the analysis of quantitative data, followed by the analysis of qualitative data to explain the results in more depth [63]. The visual diagram of our study design is shown in Fig 1.

### Quantitative phase

#### Data collection.

We used data from a longitudinal dengue study designed and implemented by our EcoDess research team in Esmeraldas, Ecuador. This study conducts annual household surveys to capture information regarding piped water supply and coping strategies to deal with water intermittency using household surveys. For this cross-sectional analysis, we used data collected in the 2021 household survey, which took place between April 8 and September 13, 2021. In cases where households did not have data available for 2021, we used data from the 2019 household survey, which was conducted between August 14 to October 2, 2019. No surveys were conducted in 2020 due to a temporary suspension of field activities during the COVID-19 pandemic. The timing of data collection in each round depended on logistical constraints. All households in each community were eligible to enroll. For our analysis, we included only households with access to piped water, which represented 84%, 55% and 87% of households in Borbón, Maldonado and Timbiré, respectively.

To assess household piped water intermittency in the week prior to the survey, we took three approaches, all based on self-reported data: 1) weekly frequency of intermittency, defined as the number of days per week without piped water supply, 2) duration of intermittency, defined as the number of hours per day without piped water supply, and 3) weekly duration of intermittency, defined as the number of hours per week without piped water supply.

Coping strategies included whether the household: 1) purchased bottled water for drinking use (yes/no), 2) used multiple drinking water sources (yes/no), 3) shared drinking water with other households (yes/no), 4) treated their domestic water source (yes/no), based on aggregated responses indicating use of chlorine, larvicide, or other treatment methods, 5) had cisterns or elevated tanks to store water (yes/no), and 6) stored water in large containers such as plastic tanks (yes/no). We also collected data on various sociodemographic variables at the household level, including the age of the head of household, the sex of the head of household, the ethnicity of the head of household, the marital status of the head of household, household size, whether the family reported owning their home, continuity of the power supply, average household income, and the person responsible for managing water at the household.

#### Quantitative data analysis.

We calculated descriptive statistics for each community, which included frequency (%) of categorical variables and medians (interquartile range) for continuous variables. We used the Kruskal-Wallis test to identify differences in water intermittency between communities.

To describe the heterogeneity of water intermittency within communities, we focused on the town of Borbón, the only town in our study large enough to contained multiple neighborhoods. Borbón is subdivided into 13 neighborhoods, a division established by a local resident familiar with the area (S2 Fig). Due to the small number of households, the neighborhood *Barrio Chino* (only 3 households) was combined with the adjacent neighborhood, *Primero de Mayo*. To assess neighborhood-level differences in IWS patterns, we used negative binomial regression models where the measures of water intermittency were defined as count variables. Negative binomial regression was selected to address overdispersion, a condition in which the variance of the outcome exceeds its mean, violating the assumptions of a Poisson distribution. In our case, the variance of water intermittency exceeded the mean. The model was specified as follows:

$$log(\text{expected}\;\text{intermittency})=\beta_{0}+\beta_{1}\text{Neighborhood}$$

To assess whether coping strategies in response to water intermittency varied across communities, we ran separate logistic regression models for each coping strategy. In these models, community of residence was the exposure variable, and the specific coping strategy was the outcome.

$$log\left(\frac{P\;IWS\;\text{coping}\;\text{strategy}=1}{P\;IWS\;\text{coping}\;\text{strategy}=0}\right)=\beta_{0}+\beta_{1}\text{Community}$$

Where *P* IWS coping strategy = 1 represents the probability of a household using a specific coping strategy.

We identified potential confounders of the association between water intermittency and coping strategies using bivariate logistic regression models with household sociodemographics as exposure variables and coping strategies as outcomes. Variables with *p-values* below 0.20 were added into multivariable logistic regression models. Next, we assessed whether coping strategies were associated with water intermittency, adjusting for these potential confounders using the following equation.

$$log\left(\frac{P\;IWS\;\text{coping}\;\text{strategy}=1}{P\;IWS\;\text{coping}\;\text{strategy}=0}\right)=\beta_{0}+\beta_{1}\text{Community}+\beta_{2}\text{Intermittency}+\beta_{3}\text{Confounders}$$

All analyses were performed using R (ver. 4.1.0) statistical software [65]

## Qualitative phase

### Interview protocol development.

Because we conducted semi-structure interviews, we were able to ask participants about their perspectives on their water supply, their perceived reasons for why they were experiencing water intermittency, and the coping strategies they used to deal with IWS. Examples of the questions used in the in-depth interviews are detailed in S1 Table. This flexible format also allowed us to explore additional related topics that emerged during the conversations. As in other qualitative studies of water insecurity [66], we selected questions aimed at gaining insight into water-related stories and experiences from participants following the AAAQ (Availability, Accessibility, Acceptability, and Quality) framework for water as a human right. The AAAQ framework states that humans should have access to sufficient and regular water sources (Availability), physically and economically affordable (Accessibility), with adequate color, odor and taste (Acceptability) and that do not increase the health risk of those consuming it (Quality) [67].

### Qualitative data collection.

We conducted 56 in-depth semi-structure interviews during the wet season, between January 16 and January 27, 2023, with 20 interviews in Borbón, 20 in Maldonado, and 16 in Timbiré. Men and women older than 18 years old were approached through a convenience sampling approach among those present at home in the local communities during the study period. We aimed to consent a minimum of 20 participants per community, however, the final number of participants enrolled was determined based on the saturation criterion. This criterion was met when additional interviews, collected from diverse individuals, added no additional information. To ensure maximum variation, we interviewed participants from different ages, socioeconomic status (SES), and neighborhoods within each community. Conversations were audio-recorded and conducted in Spanish. Local fieldworkers were present during the interviews.

### Qualitative data analysis.

The interviews were transcribed manually and verbatim, then imported into Dedoose software for analysis [68]. We used a deductive approach to develop a codebook based on our research question and the list of questions included in the interview. Using an open coding approach, we added additional codes after examining the transcripts. Finally, we grouped codes into themes to identify patterns related to our research questions [69]. Each quote is followed by a code indicating the participant’s ID, year of interview, and community (e.g., C20–23, Borbón refers to participant 20, interviewed in 2023, in Borbón).

#### Integration of Quantitative and Qualitative Results

We used qualitative data to provide insights and contextualize our quantitative results. A joint display table [70] was created to integrate the quantitative and qualitative results to compare differences in experiences among participants.

## Results

We analyzed survey data from 1172 households, including 1089 surveyed in 2021 and an additional 83 with data available only from their 2019 household survey. Among the households, 815 were from Borbón, 183 from Maldonado, and 174 from Timbiré. Household sociodemographic characteristics are shown in Table 1.

### Water intermittency patterns differed by the community of residence

**Fig 2.**
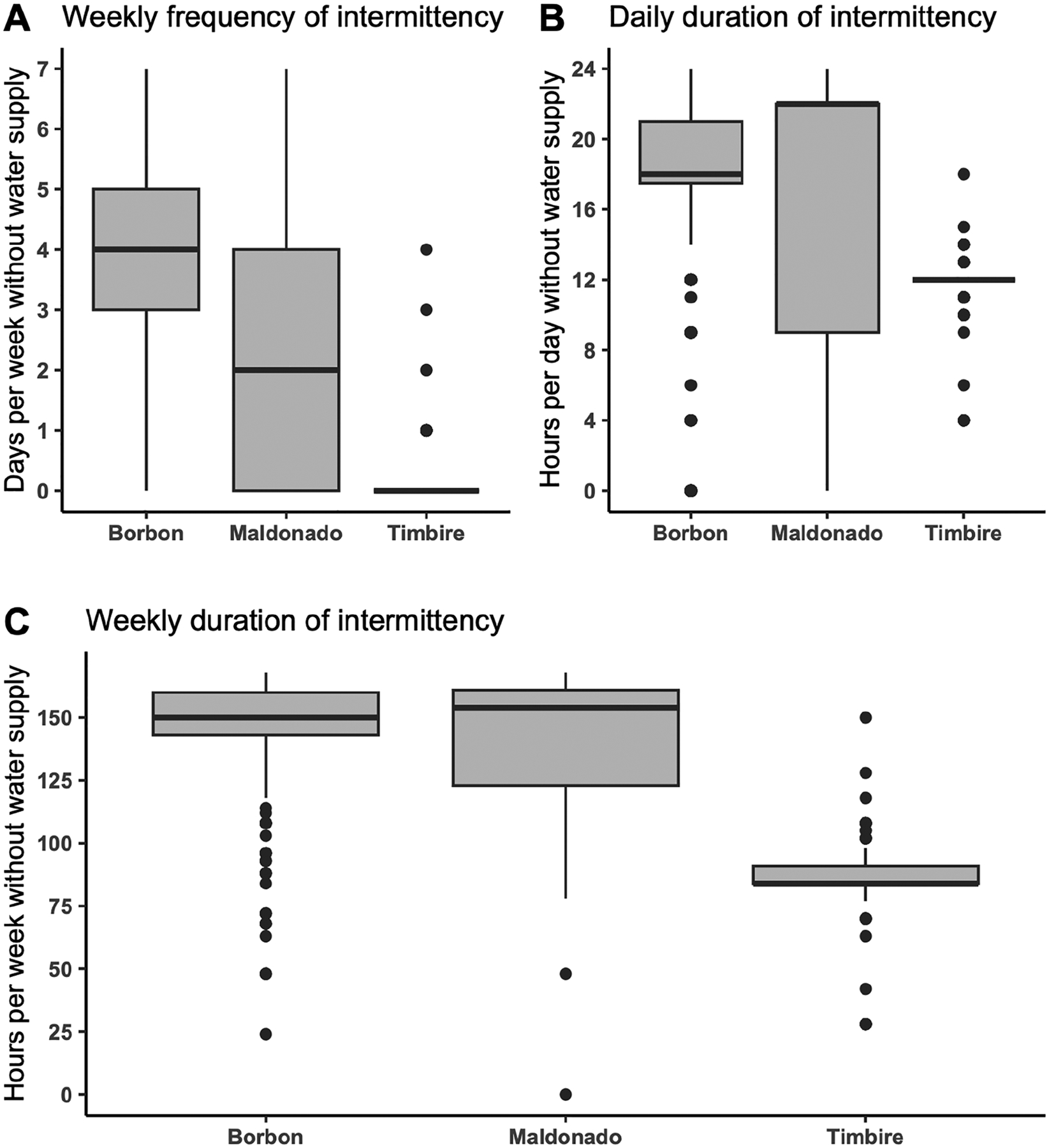
Distribution of piped water intermittency characteristics across communities. Measures of water intermittency across communities in the week prior to the survey: (a) weekly frequency of intermittency (days per week without water supply), (b) daily duration of intermittency (hours per day without water supply), and (c) weekly duration of intermittency (total hours per week without water supply). https://doi.org/10.1371/journal.pwat.0000439.g002

All three study communities experienced IWS, but the weekly frequency and daily duration of intermittent periods varied significantly by community (*p*-value: < 0.001). Borbón had the highest median number of days per week without water supply, followed by Maldonado, and then Timbiré. Conversely, Maldonado had the highest median number of hours per day without water service, followed by Borbón and then Timbiré (Fig 2).

### Predictability of water supply among communities

In-depth interviews highlighted the extent to which the unpredictability of the water system added uncertainty and caused disruption in people’s daily lives. Most participants in Borbón described their water service as unpredictable, often forcing them to pause activities to prioritize tasks that required water whenever supply became available.

“Sometimes [piped water] comes early in the morning; those who are able fill their tanks, while those who aren’t, don’t. […] sometimes it returns after a week, and sometimes it’s three days. Sometimes [it comes] in the early morning and sometimes not until noon. There is no schedule”

(C20–23, Borbón)

“Sometimes you want to do laundry, but there is no water available. When I have water, I wash clothes, we don’t let the laundry pile up. There are people that end up with piles of clothes; it’s not out of laziness, it’s because there is just no water”

(C18–23, Borbón)

In Maldonado and Timbiré, participants described their water service as mostly predictable. In Maldonado, participants explained that piped water was supplied twice a day for short durations. Consequently, residents made preparations within that time frame, such as waking up early or planning to be at home when supply was expected:

“There are days when they pump [piped water] in the morning, twice a day, I mean, they pump it from 6 to 7am and then from 9 to 10am. But at my house, sometimes I can’t even manage to fill up one tank of water”

(C35–23, Maldonado)

“If you run out of water today, you have to wait until tomorrow, or else you have to buy water or go to the river”

(C39–23, Maldonado)

In Timbiré, participants expected to receive water daily, indicating a level of predictability in the supply. While occasional disruptions occurred, these were exceptions rather than the norm:

“We get water every day, […] they cut off the supply only when something at the plant breaks or when they’re doing maintenance”

(C55–23, Timbiré)

### Neighborhood-level differences in IWS patterns within the large town of Borbón

Neighborhoods within Borbón (S2 Fig) varied by frequency and duration of intermittent periods; however, adjacent neighborhoods experienced similar IWS characteristics. For example, the adjacent neighborhoods of *Primero de Mayo*, *Torres Gemelas*, and *Nuevo Amanecer* had over 90% higher risk of reporting more days/week without water supply compared to *Y de Huevito*, the closest neighborhood to the local water plant (Table 2). Sociodemographic variables, which also varied by neighborhood of residence, are present in S2 Table to provide additional context.

### Household coping strategies vary depending on IWS patterns

Coping strategies to deal with water intermittency varied across the three communities. Households in Maldonado (characterized by low frequency but long duration of water intermittency) were more likely to purchase bottled water and treat their domestic water, but less likely to use multiple drinking water sources compared with Borbón (characterized by medium frequency and duration of water intermittency). On the other hand, households in Timbiré (characterized by low frequency and duration of water intermittency) were less likely to treat their domestic water, but more likely to use multiple drinking water sources and share water compared to households in Borbón. Moreover, households in Timbiré were less likely to store water in large containers and have cisterns/elevated tanks (Table 3). Observed frequencies of coping strategies in each community are provided in S3 Table.

Household sociodemographic factors were associated with coping strategies. For instance, households with an average income above the national minimum wage were more likely to purchase bottled water, have cisterns or elevated tanks, and store water in large containers compared to households with lower incomes. Other sociodemographic factors associated with coping strategies included ethnicity of the head of household, household size, and the individual responsible for water management (S4 Table). After adjusting for these potential confounders, each 1-day increase in water intermittency was associated with a 30% increase in the odds of treating domestic water, while the odds of using multiple drinking sources decreased by 18%. Higher weekly frequency was also associated with an increased likelihood of purchasing bottled water, although this association was not statistically significant. In addition, for each 3-hour increase in the duration of intermittent periods and weekly intermittency, the odds of treating domestic water increased by 12% and 4%, respectively. There was no evidence of a significant association between intermittency and sharing drinking water, having a cistern or elevated tank and storing water in large containers (Table 4).

### In-depth interviews provided context for coping strategies and other water-related behaviors

#### Using multiple drinking water sources.

The use of multiple drinking water sources varied due to the perceived risks associated with piped water. Households in Borbón and Maldonado predominantly relied on bottled water, specifically 20-liter reusable bottles, as they did not trust their water system. Secondary sources for drinking purposes, such as piped water or rainwater, were only considered a last resort when bottled water was not available. In contrast, most households in Timbiré perceived piped water as safe and fit for consumption, using piped water alongside bottled water.

Many participants (especially in Maldonado) referred to piped water being unsafe for consumption due to the presence of sediments and rust residues, as assessed through sight, smell, and taste. Participants also mentioned how they would assess levels of water contamination by observing changes in the appearance of food when cooking with piped water. For example, the color of plantains would turn from yellow to black. As a result, participants from both Borbón and Maldonado considered bottled water as the safest and most convenient source of drinking water available. Although most participants were not certain about the source, purification process, and packaging components of bottled water, and its cost was a concern for many, the poor quality of their local piped water left them no choice but to rely on bottled water. Participants appreciated the convenience of the established distribution network that delivered bottled water directly to their households or to nearby grocery stores.

#### Sharing water sources.

Although regular water sharing between households was not frequent, with only three participants mentioned doing so consistently, residents from all communities described experiences when they were willing to help another neighbor or family member when they needed water. For some participants, social networks and relationships played a critical role in facilitating water sharing between households.

#### Storing water.

All interviewees stored water to ensure they had enough for their daily needs, as unexpected shortages were frequent. In Borbón, where households might go days without water supply, storing water in large containers ensures water for the days ahead. In contrast, in Maldonado, where most households receive water every day but for only a few hours, storing water ensured water for the hours ahead. Participants in Maldonado mentioned refilling their containers daily, preventing water from remaining stagnant for extended periods of time. In Timbiré, participants used a mix of large containers and smaller ones like buckets and pots to store water.

Having a cistern or an elevated tank provided large storage capacity and convenience when the storage was directly connected to the household water pipes, mimicking a continuous supply. However, such systems required a significant investment, making them unaffordable for many households in the area.

#### Domestic water treatment.

In Borbón and Maldonado, participants described relying on stored water on the days or hours when they did not receive a water supply and how they treated this water to prevent the growth of organisms, particularly mosquito larvae, which they saw as a major health concern. Treatment methods included adding chlorine, typically purchased by households, and larvicides, provided by government-run malaria control programs. In Timbiré, participants noted a strong chlorine smell in their piped water, indicating it had already been treated, so no additional treatment was needed.

#### Other coping strategies identified through in-depth interviews.

Participants brought up additional coping strategies not captured in our surveys, including minimizing water use during periods of intermittency. They described prioritizing water for personal hygiene needs, such as cleaning intimate areas, while avoiding using piped water for tasks like washing clothes or dishes.

“The thing is, we are always on alert. If we see that our water supply is running low, then we cut back our consumption. We reduce it, we try to reduce it to avoid running completely out of water.”

(C17–23, Borbón)

“For washing dishes, for example, I use two containers, one for soaping up the dishes and the other for rinsing them off.”

(C20–23, Borbón)

Another coping strategy frequently used by households was recycling greywater. Participants mentioned reusing the final rinse water from washing clothes, which often contains chlorine or detergent residues, for household cleaning or mopping.

“When you rinse clothes, we use [that water] for the bathroom, for cleaning the house… when you wash the dishes, we use the water too and pour it into the bathroom”

(C18–23, Borbón)

Participants mentioned the advantages of investing in water pumps and connecting them to their pipes to extract additional water during supply periods, especially when the supply has low pressure. These included both portable and non-portable motorized pumps, which are used to draw water more effectively when connected to the piped system. However, it was also noted that the use of pumps can lead to negative consequences for those who cannot afford them, as they can drain the available water supply in their immediate vicinity.

“Most people don’t get a good flow of water because of the pump, it pulls too much water and then those with large cisterns can fill up. […] Well, this affects most people… […] They don’t get a strong water flow; the water barely reaches them. And when they want to fill up, they can’t get much because the water flow just isn’t there”

(C39–23, Maldonado)

### Integration of quantitative and qualitative findings

Households with more frequent intermittency were less likely to use multiple drinking water sources. Qualitative insights help explain this pattern: participants in Maldonado and Borbón reported a lack of trust in piped water, prompting them to depend more exclusively on bottled water for drinking. This perceived unreliability appeared to discourage households from seeking alternative sources unless absolutely necessary. Secondary water sources were typically used only when bottled water was unavailable. Although our quantitative analysis did not find a statistically significant association between water intermittency and purchasing bottled water, the point estimate suggested a similar trend, indicating convergence with the interviews.

Households were also more likely to treat their domestic water source in response to intermittency. Interviewees described this behavior as often driven by visible signs of water contamination, such as turbidity and the presence of mosquito larvae, which they observed in stored water. Intermittency led households to store water for later use, which allow them to observe its condition more closely. These visible cues appeared to serve as a proxy for water quality, prompting households to treat their water.

A joint display table integrating qualitative and quantitative results, along with illustrative quotes is provided in S5 Table.

## Discussion

Although water intermittency was a common experience for all households in the study communities, the weekly frequency, daily duration, and predictability of intermittency varied, resulting in different household coping strategies. Households that experienced frequent episodes of intermittency in the week were more likely to treat their domestic water source, but less likely to use multiple drinking water sources. Water predictability, perceptions of safety and quality, convenience, and cost also influenced how households respond to IWS. Here we discuss each of these main findings.

All study communities, as well as neighborhoods within Borbón, exhibited distinct weekly frequencies and daily durations of intermittency. Differences between communities might be attributable to variations in system capacities, infrastructure maintenance, and the availability of water resources within each of those communities [11]. Consistent with previous findings in sub-Saharan Africa and South Asia, our results also highlight that in larger communities, IWS patterns can vary significantly by neighborhood [32,37]. Geographical factors such as elevation and proximity to water treatment plants likely impact IWS patterns within communities [11,32–35]. Recognizing both between- and within-community variability, and the factors that shape it, is essential for guiding future research on water access.

Different measures of water intermittency can lead to varying interpretations of the severity and characteristics of intermittency. Communities with similar weekly water service hours may face significantly different challenges, leading them to adopt different coping strategies to overcome IWS. Consistent with other studies [37], a combined measure of weekly duration of intermittency (hours/week) masked some of the associations related to weekly frequency (days/week) or daily duration (hours/day) of intermittency. By separately assessing the weekly frequency and daily duration of intermittent periods, we gained a more detailed understanding of disparities in water access within and between communities.

In addition to frequency and duration of intermittent periods, predictability also varied among our study sites. Predictable water systems, as reported in other studies [25], allow households to strategically prepare for water outages, such as waking early for scheduled supply. Greater predictability has been associated with a 25% reduction in emotional distress and a 50% reduction in stressful behaviors [71]. In contrast, unpredictable water systems cause disruptions in people’s routines, causing residents to pause activities to prioritize tasks that require water when the supply is available. This unpredictability has been associated with increased water-related stress and anxiety, in addition to being a known risk factor for enteric and vector-borne diseases such as dengue [72]. Measuring water supply frequency and duration does not provide information about the predictability of the system. Since predictability affects how households cope with water intermittency, future work should collect data on predictability of water supply and explore its potential to mitigate the adverse effects of unreliable water systems. Understanding this relationship could lead to better strategies for water management in settings where continuous water supply is not possible.

Previous studies have reported the use of multiple water sources to meet household water needs during breakdowns or IWS [48,73]. When possible, low-income communities often turn to rainwater due to its abundance and convenience [73]. For instance, in rural Mexico, most households collected rainwater for drinking purposes and supplemented this source with piped water, well water, surface water and bottled water [74]. In our study setting, however, households with more frequent intermittency were less likely to report using multiple drinking water sources. One possible explanation is that unreliable water systems erode trust in piped water, prompting households to depend more exclusively on bottled water to fulfill their drinking needs. While the association between intermittency and purchasing bottled water was not statistically significant a shift toward reliance on bottled water is consistent with findings from other studies. In Indonesia, most households relied on refilled water from kiosks instead of piped water due to concerns about water quality [75]. Similarly, in urban Ghana, 44% of households relied on sachet water as their primary drinking source, using secondary sources such as rainwater or stored water only when sachet water was unavailable [76]. Given that secondary, unimproved water sources are typically at a higher risk of fecal contamination compared with improved sources [4], the sporadic use of these sources, even if only for a few days each year, may undermine the health benefits from using safe drinking water [77].

Water storage has extensively been reported as a coping strategy for IWS [25,46,49], and is a known risk factor for microbial exposure due to contamination during transportation or recontamination events when managing stored water [20,78–80]. In our study area, there was no evidence of a significant association between water intermittency and the household’s likelihood of storing water in large containers. This is likely because most households stored water in large containers, regardless of their water supply, potentially obscuring the identification of distinct patterns. However, our qualitative results indicate that the rate of turnover of stored water was likely influenced by intermittency. Consumers often store as much water as possible due to fear of shortages. Any unused stored water is replaced with fresh water during the next delivery. These behaviors have key implications for research understanding the health risks of IWS, as surveys focusing only on the volume of stored water may overlook turnover rates and treatment practices.

Quantitative and qualitative studies have identified household water treatment as a common coping strategy in response to suboptimal water quality in intermittent settings [45,54]. In our study, in-depth interviews suggest that this behavior was often driven by visible signs of water contamination. Similar patterns have been observed in Bandung, Indonesia, where residents reported yellowing of clothes from piped water and responded by filtering or chlorinating their water [81]. In Abuja, Nigeria, households treated their water after noticing muddy supply [82]. These findings suggest how water intermittency may not only affect access but also influences perceptions of safety.

Access to some coping strategies is not necessarily equally distributed across households and often reflects broader socioeconomic inequalities. For example, we observed that households with greater financial resources were more likely to invest in infrastructure to mitigate water intermittency, such as having cisterns, elevated tanks, and large containers to store water. While no specific cost estimates were provided, and we did not systematically ask all participants about the feasibility of installing infrastructure, these insights emerged organically during the interviews. A similar pattern was reported in Kabul, Afghanistan, where low-income households primarily relied on small plastic containers for water storage, while wealthier households could afford larger tanks [83]. A recent systematic review found that higher income households were twice as likely to invest in water treatment compared to those with lower incomes [84]. Education and higher level of knowledge have also shown associations with coping strategies. Having a formal education has been associated with twice the likelihood of treating water [84] and having a secondary education in Oromia, Ethiopia was associated with 60% higher likelihood of using water treatment [85]. Gender dynamics also play a role, as women are often responsible for household water management and may bear a disproportionate burden in choosing household coping strategies [86]. Addressing the effects of IWS should be prioritized in these vulnerable populations to avoid exacerbating existing inequalities.

The explanatory sequential mixed-method design of this study enabled us to integrate participant perspectives with quantitative data, improving our interpretation of results and capturing the nuanced impacts of water intermittency. In-depth interviews allowed us to explore additional coping mechanisms not initially assessed in our surveys, including cutting back on daily water use to avoid running out of water [12,25,37], recycling grey water [25,45,50] and using pumps to get higher volumes of water when supply was available [25,75]. However, biases are inherent in our self-reported assessment of IWS. For example, participants reported the water supply schedule they were aware of, potentially differing from actual distribution periods. Factors like gender, SES, or the presence of a household cistern that provides a continuous water source even when the supply is interrupted, might cause households to be more aware of and concerned about IWS than others. Future studies should supplement self-reported data with objective measures of IWS obtained directly from water service providers if available. Furthermore, although it is well established that household adoption of coping strategies can vary seasonally [76], our ability to examine the role of seasonality was limited due to the cross-sectional design of our study. Most household data were collected during the dry season (April to September 2021), while in-depth interviews took place during the wet season (January 2023). As a result, reported experiences of water intermittency may reflect different seasonal contexts, which could affect comparability across methods. Finally, although we did not find a statistically significant association between intermittency and bottled water use for drinking, this may be due to limited sample size. Future studies with larger sample are needed to clarify this relationship.

## Conclusion

Providing households with a piped water supply has long been considered an improved water system, with the assumption that piped water is associated with lower health risks. However, over the past two decades, it has become clear that inadequately designed water systems can result in unreliable and potentially contaminated water. Through a combination of statistical modeling and qualitative analysis, our findings suggest that IWS prompt households to adopt different coping strategies, each with its own associated cost, and that the type of water intermittency (weekly frequency, daily duration, and predictability) is an important determinant of the specific coping strategies selected. These coping strategies introduce additional risks associated with IWS. Ultimately, although appropriately designing and maintaining a water system is complex and requires a thorough understanding of social, ecological and political processes, learning from successful water systems can help improve less successful ones. Improvements will require engineering expertise, government commitment to investing in underserved towns, and engagement with community members to understand their experiences, constraints, and values.

## Supplementary Material

S2 Fig. Distribution of neighborhoods in the town of Borbón. **S2 Fig. Distribution of neighborhoods in the town of Borbón.** The town of Borbón is subdivided into 13 neighborhoods, a division established by a local resident familiar with the area. The local water plant is located to the west, adjacent to the neighborhood “Y de Huevito”. The map was plotted using the free and open-source software QGIS, version 3.34.3-Prizren, available for download at https://qgis.org/download/, with the Retina Tiles basemap (accessed via the XYZ Tiles feature).
(TIFF)

S1 Table. Examples of qualitative questions used in the in-depth semi-structured interviews. **S1 Table. Examples of qualitative questions used in the in-depth semi-structured interviews.** In-depth semi-structured interviews included questions aimed at gaining insight into water-related stories and experiences from participants following the AAAQ (Availability, Accessibility, Acceptability, and Quality) framework for water as a human right. Additional related topics that emerged during the conversations were also explored.
(XLSX)

S2 Table Household sociodemographic characteristics by neighborhood in Borbón. **S2 Table Household sociodemographic characteristics by neighborhood in Borbón.** This table presents the frequencies and percentages of household-level sociodemographic characteristics by neighborhood in Borbón. For reference, the Ecuadorian national wage in 2019 was $394 USD per month.
(XLSX)

S3 Table Frequency of coping strategies by community of residence. **S3 Table Frequency of coping strategies by community of residence.** This table presents the frequencies and percentages of coping strategies used by household to deal with water intermittency. We classified the variable ‘Domestic water treatment’ (yes/no), based on aggregated responses indicating use of chlorine, larvicide, or other treatment methods.
(XLSX)

S4 Table. Multivariable negative binomial regression models assessing the association between household sociodemographics and coping strategies. **S4 Table. Multivariable negative binomial regression models assessing the association between household sociodemographics and coping strategies.** Household-level sociodemographic characteristics that were statistically associated with coping strategies (*p-values* < 0.20) in bivariate logistic regression models were added into multivariable logistic regression models. Variables that remained significant were added as confounders in the models evaluating the association between piped water intermittency and coping strategies presented in Table 4.
(XLSX)

S1 Fig. Map of study communities in northwestern Ecuador included in this analysis, categorized by remoteness level. **S1 Fig. Map of study communities in northwestern Ecuador included in this analysis, categorized by remoteness level.** Our study site included three communities in northwestern Ecuador. Borbón (population ~7000), the region’s main commercial center, is connected by secondary roads with Maldonado (pop. ~ 2000) and Timbiré (pop. ~ 1000), located 20 and 40 minutes away, respectively. The map was plotted using the free and open-source software QGIS, version 3.34.3-Prizren, available for download at https://qgis.org/download/, with the Retina Tiles basemap (accessed via the XYZ Tiles feature).
(TIFF)

S5 Table. Display of participant experiences for different coping strategies. **S5 Table. Display of participant experiences for different coping strategies.** In this table we present the joint display table integrating qualitative and quantitative results, along with illustrative quotes.
(XLSX)

## Figures and Tables

**Table 1.** Household characteristics by community of residence.

		Total		Borbón		Maldonado		Timbiré	
		(n = 1172)		(n = 815)		(n = 183)		(n = 174)	
		Frequency	Percentage	Frequency	Percentage	Frequency	Percentage	Frequency	Percentage
Water Intermittency	Days/week without water supply*	3 [1–4]	---	4 [3–5]	---	2 [0–4]	---	0 [0–0]	---
	Hours/day without water supply*	18 [14–21]	---	18 [17–21]	---	22 [9–22]	---	12 [12–12]	---
	Hours/week without water supply*	147 [123–159]	---	150 [143–160]	---	154 [123–140]	---	84 [84–91]	---
Age	<36 years	257	21.9%	174	21.3%	43	23.5%	40	23.0%
	36–46 years	309	26.4%	210	25.8%	44	24.0%	55	31.6%
	47–60 years	322	27.5%	239	29.3%	42	23.0%	41	23.6%
	>60 years	284	24.2%	192	23.6%	54	29.5%	38	21.8%
Sex of head of household	Male	712	60.8%	491	60.2%	113	61.7%	108	62.1%
	Female/Other	460	39.2%	324	39.8%	70	38.3%	66	37.9%
Ethnicity of head of household	Afro-Ecuadorian	840	71.7%	534	65.5%	145	79.2%	161	92.5%
	Indigenous	32	2.7%	28	3.4%	4	2.2%	0	0.0%
	Mestizo/Other	300	25.6%	253	31.1%	34	18.6%	13	7.5%
Marital status of head of household	Single	374	31.9%	253	31.0%	59	32.2%	62	35.6%
	Married/living together	679	57.9%	462	56.7%	112	61.2%	105	60.3%
	Divorced/separated/widowed	119	10.2%	100	12.3%	12	6.6%	7	4.1%
Household size	Small (up to 3)	593	50.6%	405	49.7%	96	52.5%	92	52.9%
	Medium (4–6)	493	42.1%	346	42.5%	75	41.0%	72	41.4%
	Large (at least 7)	86	7.3%	64	7.8%	12	6.5%	10	5.7%
House ownership	No	195	16.6%	127	15.6%	43	23.5%	25	14.4%
	Yes	977	83.4%	688	84.4%	140	76.5%	149	85.6%
Power supply	Interrupted	600	51.2%	353	43.3%	148	80.9%	99	56.9%
	Continuous	572	48.8%	462	56.7%	35	19.1%	75	43.1%
Average household income	<$394	385	32.8%	203	24.9%	39	21.3%	143	82.2%
	> $394	315	26.9%	246	30.2%	39	21.3%	30	17.2%
	Skipped	472	40.3%	366	44.9%	105	57.4%	1	0.6%
Water responsible	Adult man/other	618	52.7%	460	56.4%	66	36.1%	92	52.9%
	Adult woman	554	47.3%	355	43.6%	117	63.9%	82	47.1%

Household characteristics by community of residence. For reference, the Ecuadorian national wage in 2019 was $394 USD per month.

* Median [IQR].

https://doi.org/10.1371/journal.pwat.0000439.t001

**Table 2.** Negative binomial regression models assessing the association between neighborhood of residence in Borbón and different measures of water intermittency (n = 814).

Neighborhood	N (%)	Weekly frequency IWS		Daily duration IWS		Weekly duration IWS	
		Incidence Rate Ratios	*p*-value	Incidence Rate Ratios	*p*-value	Incidence Rate Ratios	*p*-value
Y de Huevito (ref)	44 (5.4)	---	---	---	---	---	---
Miduvi	59 (7.2)	1.1 [0.9 – 1.6]	0.37	1.0 [0.9 – 1.2]	0.71	1.0 [1.0 – 1.1]	0.45
Lumber	28 (3.4)	1.2 [0.9 – 1.5]	0.34	1.0 [0.9 – 1.2]	0.77	1.0 [1.0 – 1.1]	0.49
Niño Jesus	13 (1.6)	1.3 [0.8 – 1.9]	0.23	0.9 [0.8 – 1.1]	0.33	1.0 [0.9 – 1.1]	0.59
Primero de Mayo	41 (5.0)	2.0 [1.5 – 2.6]	**<0.001**	1.1 [1.0 – 1.3]	0.16	1.1 [1.0 – 1.2]	**0.003**
Torres Gemelas	84 (10.3)	1.9 [1.5 – 2.5]	**<0.001**	1.1 [1.0 – 1.3]	**0.036**	1.1 [1.1 – 1.2]	**<0.001**
Nuevo Amanecer	15 (1.8)	2.0 [1.4 – 2.7]	**<0.001**	1.1 [0.9 – 1.3]	0.52	1.1 [1.0 – 1.2]	**0.016**
Cayapas	112 (13.8)	1.7 [1.3 – 2.2]	**<0.001**	1.0 [0.9 – 1.2]	0.50	1.1 [1.0 – 1.2]	**0.002**
Estación	134 (16.5)	1.7 [1.3 – 2.2]	**<0.001**	1.1 [1.0 – 1.3]	**0.027**	1.1 [1.1 – 1.2]	**<0.001**
Cinco de Agosto	150 (18.4)	1.5 [1.2 – 2.0]	**0.002**	1.0 [0.9 – 1.1]	0.38	1.0 [1.0 – 1.1]	0.69
Lechugal	103 (12.7)	1.7 [1.3 – 2.2]	**<0.001**	0.9 [0.8 – 1.1]	0.35	1.0 [1.0 – 1.1]	0.23
Malecón	31 (3.8)	1.4 [1.0 – 1.9]	**0.06**	0.8 [0.7 – 1.0]	**0.014**	0.9 [0.9 – 1.0]	**0.038**

Incidence rate ratios greater than 1 indicate that the neighborhood reported more intermittency compared to the reference neighborhood (*Y de Huevito*). Missing value: Neighborhood information (n = 1).

https://doi.org/10.1371/journal.pwat.0000439.t002

**Table 3.** Logistic regression model assessing the association between coping strategies and community (n = 1172).

Community	Purchase bottled water for drinking use		Multiple drinking water sources		Share drinking water		Domestic water treatment		Cistern/elevated tank		Water storage in large containers	
	OR [95% CI]	*P*-value	OR [95% CI]	*p*-value	OR [95% CI]	*p*-value	OR [95% CI]	*p*-value	OR [95% CI]	*p*-value	OR [95% CI]	*p*-value
Borbón (ref)	---	---	---	---	---	---	---	---	---	---	---	---
Maldonado	1.96 [1.11 – 3.72]	**0.03**	0.16 [0.08 – 0.30]	**<0.001**	0.74 [0.04 – 4.37]	0.78	1.89 [1.37 – 2.62]	**<0.001**	1.07 [0.64 – 1.73]	0.78	0.86 [0.56 – 1.36]	0.51
Timbiré	0.02 [0.01 – 0.03]	**<0.001**	87.25 [41.48 – 224.56]	**<0.001**	3.99 [1.14 – 13.39]	**0.02**	0.03 [0.01 – 0.08]	**<0.001**	0.09 [0.01 – 0.29]	**0.001**	0.13 [0.09 – 0.18]	**<0.001**

Odds ratios greater than 1 indicate that the community is more likely to use the specific coping strategy compared to the reference community, Borbón.

https://doi.org/10.1371/journal.pwat.0000439.t003

**Table 4.** Multivariate logistic regression model assessing the association between coping strategies and water intermittency (n = 1172).

Water intermittency	Purchase bottled water for drinking use		Multiple drinking water sources		Share drinking water		Domestic water treatment		Cistern/elevated tank		Water storage in large containers	
	OR [95% CI]	*p*-value	OR [95% CI]	*p*-value	OR [95% CI]	*p*-value	OR [95% CI]	*p*-value	OR [95% CI]	*p*-value	OR [95% CI]	*p*-value
Weekly frequency of intermittency (days/week)	1.13 [0.99 – 1.29]	0.08	0.82 [0.72 – 0.92]	**0.001**	1.18 [0.75 – 1.83]	0.46	1.30 [1.18 – 1.41]	**<0.001**	1.01 [0.88 – 1.15]	0.90	1.09 [0.98 – 1.22]	0.13
Daily duration of intermittency (hours/day) +	1.01 [0.96 – 1.05]	0.79	1.01 [0.90 – 1.15]	0.84	0.90 [0.66 – 1.36]	0.55	1.12 [1.04 – 1.22]	**0.005**	1.01 [0.91 – 1.14]	0.81	1.03 [0.93 – 1.14]	0.52
Weekly duration of intermittency (hours/week) +	1.00 [0.97 – 1.03]	0.77	0.98 [0.96 – 1.01]	0.14	0.96 [0.91 – 1.03]	0.16	1.04 [1.02 – 1.06]	**<0.001**	1.00 [0.98 – 1.03]	0.95	1.00 [0.98 – 1.02]	0.90

Odds ratios greater than 1 indicate that households with higher intermittency are more likely to use the specific coping strategy. Models are adjusted for the community of residence and relevant sociodemographic variables. The specific covariates vary by the outcome (coping strategy) as follows: Purchase bottled water for drinking use (ethnicity of head of household, power supply and average household income), Multiple drinking water sources (ethnicity and marital status of head of household, household size, power supply and average household income), Share drinking water (household ownership), Domestic water treatment (marital status of head of household, power supply, average household income and water responsible), Cistern/elevated tank (age and ethnicity of head of household, household size, and average household income), Water storage in large containers (household size, average household income and water responsible).

+ Models show the effect of a 3-unit increase in duration of water intermittency.

https://doi.org/10.1371/journal.pwat.0000439.t004

## Data Availability

The data and R scripts supporting the findings of this study are publicly available on GitHub at [https://github.com/arsosa/WaterIntermittency_CopingStrategies].
