# Supplementary figures and images for: How water intermittency and water perceptions influence household coping strategies in northwestern Ecuador
Source: PLOS Water. Author manuscript; Available in PMC 2026 Feb 28. (PMC12948165; doi:10.1371/journal.pwat.0000439)

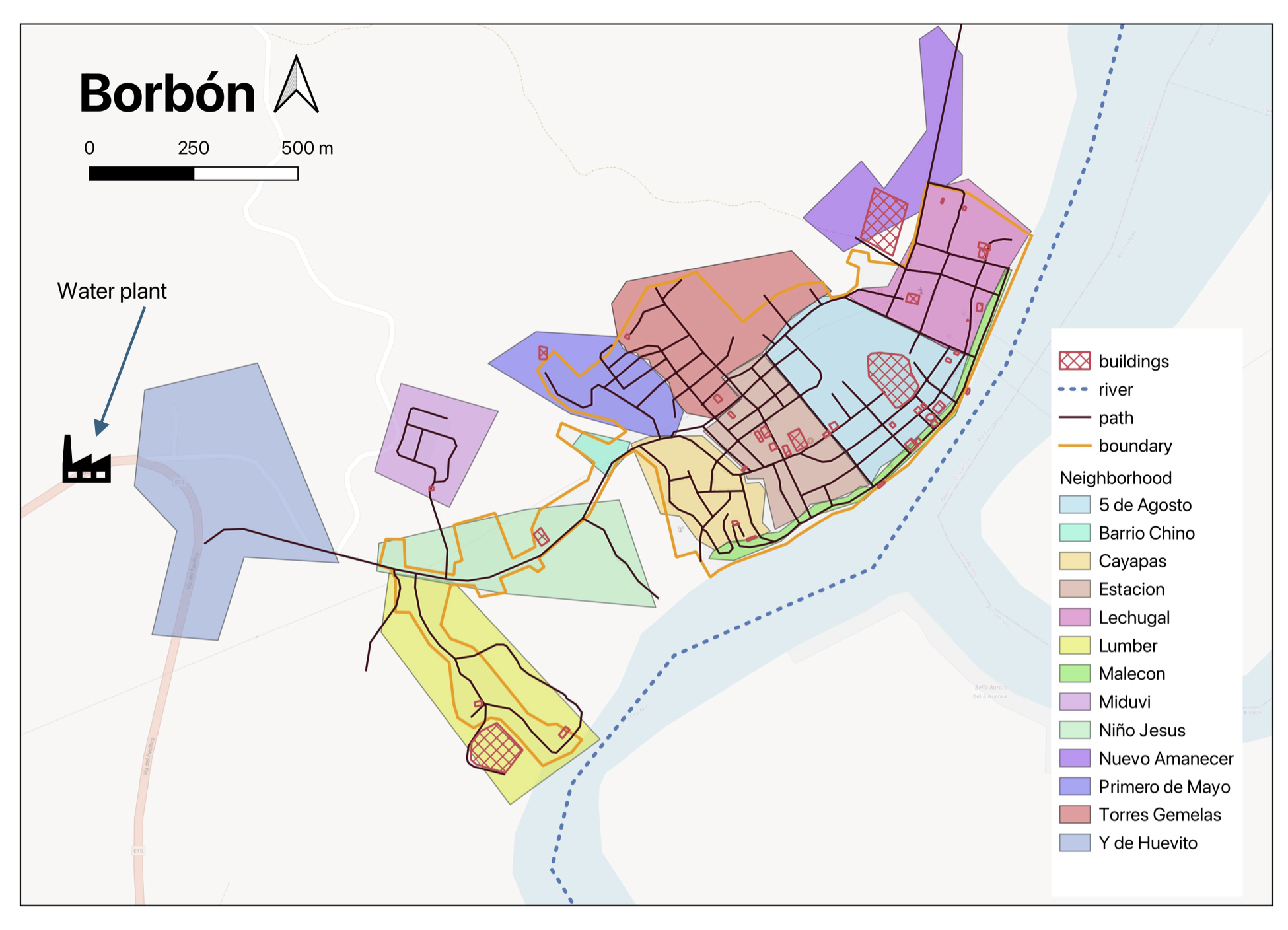

Supplement: S2 Fig. Distribution of neighborhoods in the town of Borbón. — The town of Borbón is subdivided into 13 neighborhoods, a division established by a local resident familiar with the area. The local water plant is located to the west, adjacent to the neighborhood “Y de Huevito”. The map was plotted using the free and open-source software QGIS, version 3.34.3-Prizren, available for download at https://qgis.org/download/, with the Retina Tiles basemap (accessed via the XYZ Tiles feature). (TIFF) [file NIHMS2143460-supplement-S2_Fig__Distribution_of_neighborhoods_in_the_town_of_Borb_n_.tiff]

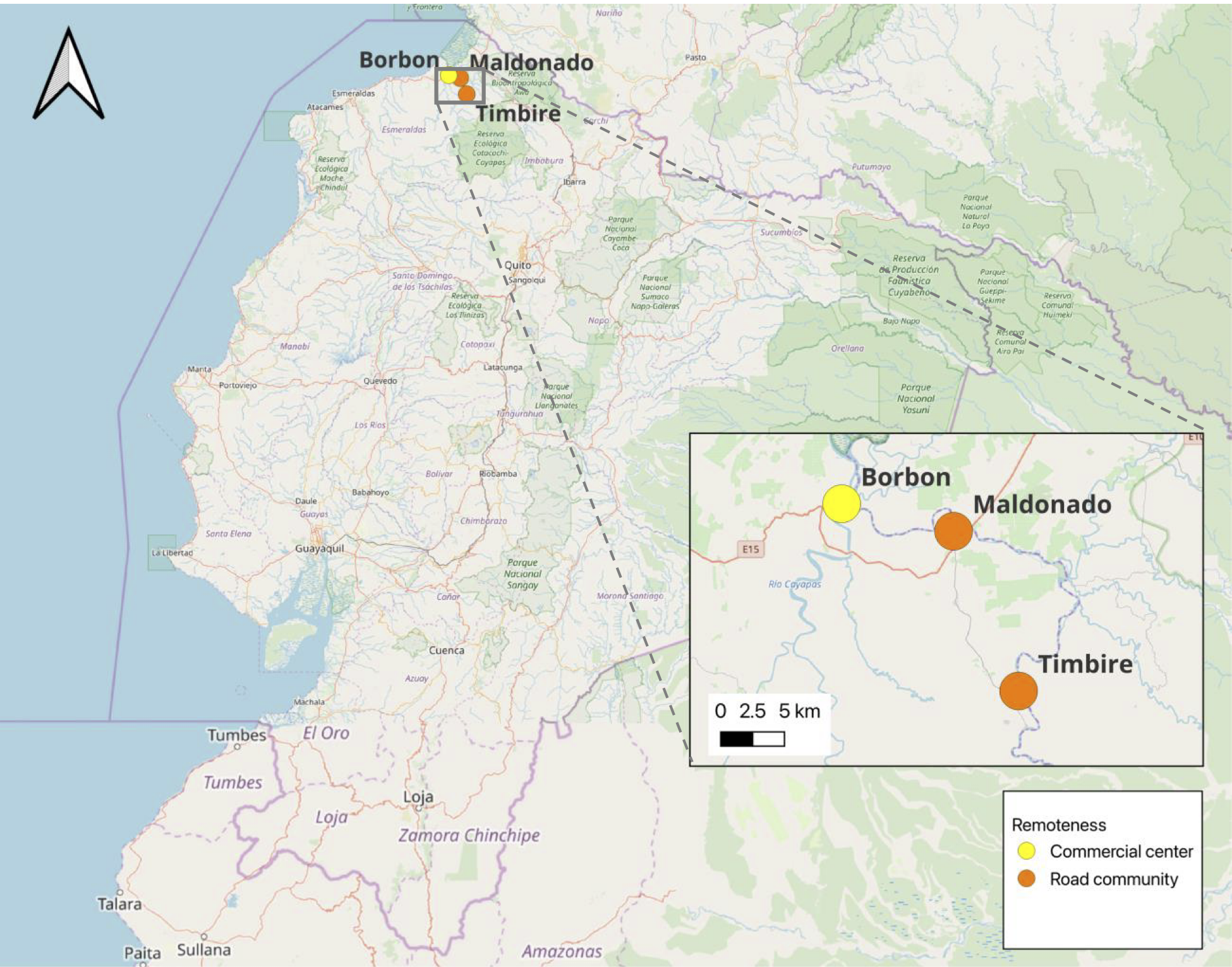

Supplement: S1 Fig. Map of study communities in northwestern Ecuador included in this analysis, categorized by remoteness level. — Our study site included three communities in northwestern Ecuador. Borbón (population ~7000), the region’s main commercial center, is connected by secondary roads with Maldonado (pop. ~ 2000) and Timbiré (pop. ~ 1000), located 20 and 40 minutes away, respectively. The map was plotted using the free and open-source software QGIS, version 3.34.3-Prizren, available for download at https://qgis.org/download/, with the Retina Tiles basemap (accessed via the XYZ Tiles feature). (TIFF) [file NIHMS2143460-supplement-S1_Fig__Map_of_study_communities_in_northwestern_Ecuador_included_in_this_analysis__categorized_by_remoteness_level_.tiff]
